# Gut microbial ecology and function of a Pakistani cohort with Iron deficiency Anemia

**DOI:** 10.1038/s41598-025-02556-0

**Published:** 2025-05-20

**Authors:** Hajra Khurshid, Muhammad Bilal Jamshaid, Zahra Salahuudin, Kashaf Sibtain, Iqra Fayyaz, Aqsa Ameer, Caroline Kerbiriou, Shona Mckirdy, Sobia Nawaz Malik, Konstantinos Gerasimidis, Zobia Noreen, Sundus Javed, Aafia Tariq, Umer Zeeshan Ijaz, Aamira Tariq

**Affiliations:** 1 Department of Biosciences, COMSATS University Islamabad, Islamabad, 45550 Pakistan; 2 School of Medicine, Dentistry & Nursing, Glasgow Royal Infirmary, Glasgow, G31 2ER UK; 3 Cosmesurge Hospital, Rawalpindi, Pakistan; 4 Water & Environment Research Group, Mazumdar-Shaw Advanced Research Centre, University of Glasgow, Glasgow, G11 6EW UK; 5 Department of Bioscience, Grand Asian University Sialkot, Sialkot, Pakistan; 6 Department of Molecular and Clinical Cancer Medicine, University of Liverpool, Liverpool, L69 7BE UK; 7 National University of Ireland, Galway, University Road, Galway, H91 TK33 Ireland

**Keywords:** Iron deficiency anemia (IDA), Short chain fatty acids (SCFA), Branched short chain fatty acids (BSCFA), Computational biology and bioinformatics, Microbiology

## Abstract

Iron deficiency anemia (IDA) is a major public health concern among women of reproductive age leading to high maternal mortality. Pakistan being one of the Lower –Middle income countries (LMIC) is facing this challenge tremendously. The objective of this study is to determine the impact of IDA on gut microbial diversity as well as its relationship with microbial metabolites.16 S rRNA gene profiling of healthy and IDA affected meconium samples was performed with additional meta-data collected through questionnaire. The anemia was linked to different dietary parameters through chi-square test of independence and Generalized Linear Latent Variable Model (GLLVM). Anaerobic bacterial genera such as *Coprococcus*,* Anaerovoracaceae*, strongly associated with anemia and negatively correlated with red meat and fish consumption. Moreover, these microbes positively correlated with branched short chain fatty acids (BSCFAs) production. BSCFAs have strong implications in metabolic disorders. This study provides a snapshot of how anemia modulates gut microbial diversity and microbial metabolites production which may have an impact on iron metabolism.

**Supplementary Information:**

The online version contains supplementary material available at 10.1038/s41598-025-02556-0.

## Introduction

Iron deficiency anemia is the most prevalent condition that arises from inadequate intake or absorption of nutrients, leading to impaired erythrocyte production and subsequent decline in red blood cell (RBC) count and hemoglobin (Hb) levels^1^. Despite its global prevalence, it is overrepresented in low- and middle-income countries (LMICs) such as Sub-Saharan Africa and Southeast Asia due to low dietary iron intake imposing a significant economic burden^2^. Various factors including deficiencies in iron, folic acid, vitamin B12, and vitamin A^3,4^, and genetic disorders contribute to anemia. Iron deficiency occurs when the synthesis of hemoglobin and iron-harboring enzymes is restricted by iron primarily due to malnutrition, inadequate intestinal iron absorption, consumption of iron-deprived foodstuff, or increased iron absorption by the fetus during pregnancy^5,6^. In SouthAsia, 52% of children under five (CU5) and 49% of women of reproductive age (WRA) are anemic^7^. Pakistan has the second- highest prevalence of anemia in CU5 (53%) and fourth highest among WRA (41.3%)^8^. Severe gestational anemia has been documented to be associated with low birth weight^9^, increased risk of preterm birth, and neonatal or maternal mortality^10^. Moreover, poor cognitive development, and delayed mental and motor development have been reported in babies born to anaemic mothers^4^.

Key parameters for anemia estimation includes hemoglobin (Hb) levels, hematocrit, RBCs count, mean corpuscular volume (MCV), mean corpuscular hemoglobin concentration (MCHC), and red cell distribution width (RDW), and Neutrophil-Lymphocyte ratio (NLR)^11^. In anemic patients, these values are typically lower than normal range, indicating the severity and type of anemia. MCV helps to classify microcytic, normocytic, or macrocytic anemia^12^. Haem and non-haem iron from dietary sources are absorbed in intestinal cells by employing multiple transporters and a peptide hormone i.e. hepcidin^12^. Regulation of intracellular iron is critical not only for humans but also for the diverse gastrointestinal microbial communities^13^. The lack of intestinal absorption leads to unadsorbed iron that modulates gut microbiota. The unadsorbed iron initiates inflammation by influencing *Streptococcus spp.*,* Enterococcus spp*., and *Clostridia spp*. and depletion of beneficial bacteria like *Lactobacillus*^14^. The close association of gut microbiota with anemia has postulated that gut microbes might contribute to hematopoiesis^15^. The human gut harbors an immense number of microbes termed microbiota that have a profound impact on nutrition, metabolism, and immunity^16^. Increased iron availability results in an increase in pathogenic bacteria such as *Salmonella typhimurium*,* E.coli*, and *Enterobacteriaceae* along with a reduction in the presence of beneficial ones including *Bifidobacteria*^17,18^. In iron-limiting conditions, the pathogenic microbes produce iron-scavenging molecules termed as siderophores. Apart from pathogenic bacteria, commensal bacteria including *Escherichia*^19^, *Bifidobacterium*^20^, *Enterococcus*^21^, and *Bacteroides*^22^ produce siderophores or use siderophores produced by other species to scavenge iron. Iron deficiency anemia leads to gut dysbiosis where a low abundance of *Lactobacillus*^23^, *Firmicutes*^23^, *genera like Coriobacteriaceae*^24^, and a high abundance of *Proteobacteria*^23^ *Enterobacteriaceae*, *Veillonellaceae*^24^ has been reported previously. Moreover, microbial metabolites such as short-chain fatty acids (SCFAs) including acetate, propionate, and butyrate, have been proposed to mediate iron absorption^22,25^. A decrease in butyrate-producing genera including *Roseburia*,* Coprococcus*, and *Butyricoccus* has been associated with IDA without any change in fecal butyrate levels^25^. Gut microbiota produce metabolites that suppress an important effector molecule i.e. hypoxia-inducible factor 2alpha (HIF-2 alpha) and increase the iron storage ferritin protein level resulting in decreased iron absorption by the host^26^.

The present study aims to identify the changes in the dynamic landscape of the gut of infants born to healthy and anaemic mothers, concerning the microbial communities present there. A comprehensive understanding of the impact of IDA on the gut microbiome and SCFAs will reveal the underlying features of its pathogenesis. This study revealed alteration in the microbiome in anemic meconium samples along with a shift in SCFAs signature that might lead to decreased iron absorption and consequently anemia.

## Results

### Association of anemia with anthropometric parameters

In the first step, we performed contingency analysis to find the linear dependence in the data set between the anemic status and all covariates of interest including dietary factors, sociodemographic features, complete blood count, education, pregnancies, and miscarriages with anemia. Amongst these, we found a very strong association of the covariables with between severe anemia, moderate anemia, and normal individuals. A self-administered questionnaire was designed to examine the characteristics of participants at the time of sample collection. We utilized the $\:{\chi\:}^{2}$ test of independence to identify dependencies between these characteristics, focusing primarily on the current health status of participants—categorized as

normal, moderate anemic, and severe anemic. Statistically significant relationships were further explored through Pearson residuals to discern attractors (positive associations) and repellents (negative associations) within these categories. The results are shown in the Additional file (Figs), and then summarized in Table 1 in terms of most significant attractors.

**Table 1:** *χ*^2^ test of independence between patient status and other categorical covariates considered in this study. The significant relationships are further explored in Fig. 1.

	^2^	*P*-value
**Patient status: Education**	13.350	0.101
**Patient status: Socio-economic status**	2.211	0.688
**Patient status: Diet Fruits**	8.017	0.012
**Patient status: Diet Vegetables**	1.887	0.420
**Patient status: Diet Chicken**	0.769	0.748
**Patient status: Diet Red meat**	26.487	0.000
**Patient status: Diet Milk**	1.275	0.529
**Patient status: Diet Fish**	16.049	0.004
**Patient status: Occupation**	3.888	0.248
**Patient status: Demographic Region**	2.710	0.231
**Patient status: Blood group**	10.323	0.701
**Patient status: Disease Hypertension**	0.320	1
**Patient status: Disease Diabetes**	1.171	0.522
**Patient status: Gestation**	0.695	0.850
**Patient status: Blood transfusion before delivery**	61.478	0.000
**Patient status: Gender of baby**	0.0367	0.984

## Dietary association with anemic status

The contingency analysis revealed significant dietary influences on anemia status. Consumption of red meat and fish was strongly associated with normal health status (Supplementary Figure S1). Participants who do not consume fruits are found more likely to suffer from severe anemia, while those who consume, showed healthier statuses ($\:{\chi\:}^{2}$ = 8.02; *p* < 0.05). Similarly, fish consumption was associated with normal health status, in contrast to non-consumers who tended to be moderately anemic ($\:{\chi\:}^{2}$ = 26.49; *p* < 0.05). Red meat consumption also demonstrated a positive association with normal health status, compared to those who do not consume red meat and so tended to have moderate and severe anemia ($\:{\chi\:}^{2}$ = 26.49; *p* < 0.05) (Supplementary Figure S1).

## Patient’s status and blood transfusion before delivery

Contingency analysis showed a strong association between anemia and blood transfusion. Individuals who received blood transfusions before delivery were severely anemic whereas the control individuals were devoid of it ($\:{\chi\:}^{2}$ = 61.48; *p* < 0.05). Consumption of red meat ($\:{\chi\:}^{2}$ = 4.06; *p* < 0.05) and fruits ($\:{\chi\:}^{2}$ = 10.11; *p* < 0.05) showed a negative association with blood transfusion. Individuals who do not consume red meat and fruits are more likely to have blood transfusions before their delivery and are anemic. Concerning demographic region, the analysis showed pregnant women living in a rural life setting frequently underwent blood transfusions ($\:{\chi\:}^{2}$ = 6.78; *p* < 0.05) (Supplementary Figure S2).

## Key risk factors associated with anemia

Log-binomial regression analysis was used to calculate prevalence ratios (risk factors) of moderate and severe anemia with different clinical parameters (Supplementary Table S1). Participants who were highly educated, within the Pakistani context, like holding a bachelor’s (undergraduate) degree, are at lower risk of having severe anemia. Individuals belonging to the upper and middle socioeconomic class are at lower risk of anemia as compared to the lower class plausibly due to better dietary intake. Consumption of red meat significantly reduced the risk (65%) of moderate anemia. Participants who received blood transfusion were at 137 times higher risk of anemia as compared to individuals who had not underwentne it. Fish consumption reduced the risk of severe anemia by 8%. Participants, whose blood group was AB- had 3 times higher risk of severe anemia. In contrast, participants with blood group A- are at a lower risk of severe anemia (Supplementary Table S1).

## 3) key clinical parameters that segregate between cohorts (Control, Anemia)

Random forest (RF) classifier using R’s random Forest package^27^was fitted on clinical parameters that include Marriage duration, Gravida, Miscarriage, APGAR (Appearance, Pulse, Grimace, Activity and Respiration) score 1, APGAR score 2, HCT (hematocrit-percentage of RBCs), MCV(mean corpuscular volume), MCH (mean corpuscular hemoglobin), PLT(platelet count) and MPV(mean platelet volume), with iron deficiency anemia patients. Out of all these parameters, hematological parameters including HCT, MCV, and MCH showed significant change in all the three groups. Among these parameters, HCT levels showed a strong decline in severe anemic, followed by moderate anemic patients as compared to control. MCH and MCV levels were low in the case of moderate and severe anemic individuals as compared to normal (Supplementary Figure S2A). HCT showed the highest mean decrease accuracy with a value above 75. The mean decrease Gini was high for HCT (< 30) (Supplementary Figure S2B) A confusion matrix was drawn to evaluate the correctness of the model. The confusion matrix showed that 86.36% accuracy has been achieved (Supplementary Figure S2C). This confusion matrix of our RF classifier showed that these hematological parameters can distinguish between anemic and normal individuals. These results showed that HCT is the most influential parameter to predict anemia. Our results are in line with the previous findings^28^ using hematological parameters to predict anemia apart from hemoglobin level.

### Major dietary habits patterns

UpSet plot shows co-occurrence patterns of different dietary parameters with anemia status. Individuals having moderate anemia consumed chicken, vegetables, and fruits. Individuals who consumed chicken and vegetables only in their diet suffered from severe anemia. Although anemic individuals also consume milk and fruits in suboptimal quantity. Normal healthy controls were consuming a balanced diet comprising chicken, vegetables, fruits, milk, red meat, and fish (Supplementary Figure S3).

## Microbial diversity in meconium samples

Redundancy analysis was carried out to identify the key factors that can lead to variability in the microbial community. Different variables like Status (Control; Anemic), Mother’s HB, Miscarriage, Socioeconomic status (Lower Middle Class; Middle Class; Upper Class), Weight, Diet, Demographic region (Rural; Urban) were analyzed. Miscarriages turned out to be the predominant variable that can alter microbial community by Bray Curtis, Weighted Uni-Frac, and Hierarchical meta-storms analysis (Table 2). Unweighted uni-frac analysis showed demographic region as another variable that can contribute towards microbial diversity (Table 2).

**Table 2:** Redundancy analysis with both forward and reverse selection was performed to select the most important parameters that explain variation in the community matrices. The initial set of variables considered are as follows (with those selected in the final PERMANOVA models in bold case): status (Control; Anaemic), Mother’s HB, **Miscarriage**, Socio-economic status (Lower Middle Class; Middle Class; Upper Class), Weight, Diet, **Demographic region** (Rural; Urban). Here df, SS, and F are Degree of Freedom, Sum of Squared Errors, and F Statistic, respectively.

Covariates	df	SS	*R* ^2^	F	*P*
Bray-Curtis Distance					
**Miscarriage**	**1**	**0.8378**	**0.0972**	**2.6917**	0.015 *
Residual	25	7.7813	0.9028		
Total	26	8.6191	1		
Unweighted Uni-Frac					
**Demographic_region**	**1**	**0.4808**	**0.06365**	**1.6995**	0.02 *
Residual	25	7.0721	0.93635		
Total	26	7.5529	1		
Weighted Uni-Frac					
**Miscarriage**	**1**	**0.49826**	**0.15895**	**5.4185**	0.011 *
Socio_economic_status	2	0.41234	0.13154	2.2421	0.064.
Fish	1	0.04918	0.01569	0.5348	0.609
Baby_weight	1	0.24382	0.07778	2.6516	0.084.
Residual	21	1.93105	0.61603		
Total	26	3.13465	1		
Hierarchical Meta-Storms					
**Miscarriage**	**1**	**0.07190**	**0.11536**	**3.4033**	0.027 *
Weight	1	0.04430	0.07108	2.0970	0.099.
Residual	24	0.50703	0.81355		
Total	26	0.62323	1		

Significance codes: 0 ‘***’ 0.001 ‘**’ 0.01 ‘*’ 0.05 ‘.’ 0.1 ‘’ 1

**Fig. 1:**
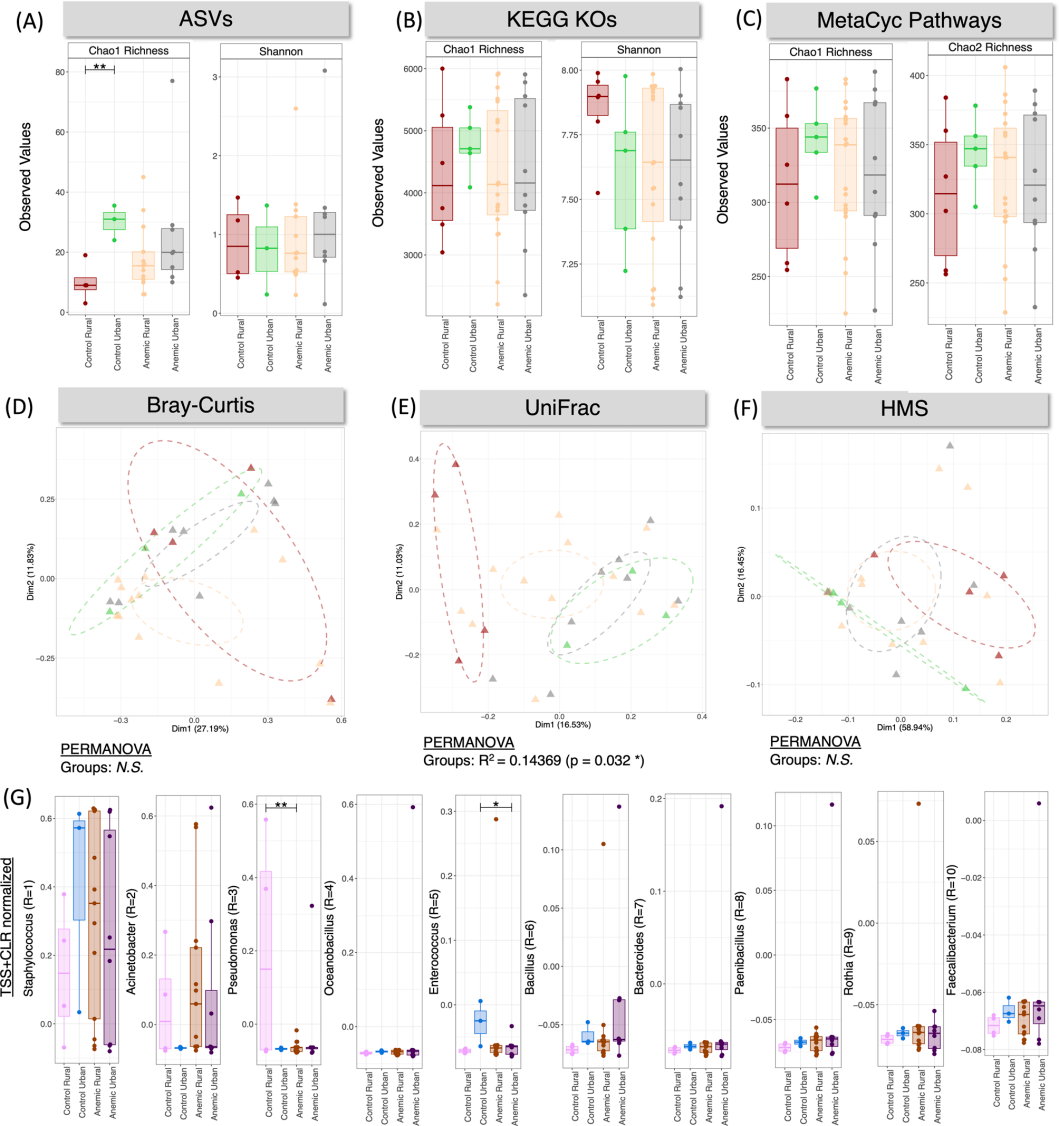
Alpha diversity (Chao 1 Richness and *Shannon* entropy) comparison of (**A**) bacterial ASVs, as well as (**B**) KEGG KOs, and (**C**) MetaCyc pathways predicted from the PICRUSt2 software. Beta diversity (**D**-**F**) represented by principal coordinate analysis (PCoA) plots with each axis showing the percentage variability explained by that axis, and where ellipses represent 95% confidence interval of the standard error for a given group. We have used three different distance measures: (**D**) *Bray-Curtis distance* to show differences in composition, (**E**) *Unweighted UniFrac distance* to show differences in phylogeny, and (**F**) *Hierarchical Meta-storms* to show differences in metabolic function. PERMANOVA statistics utilising these distance measures are shown underneath the PCoA plots to suggest if there are significant differences between the groups with R^2^ value showing percentage variability explained. (G) shows the TSS + CLR normalized expressions of top 10 most abundant genera observed in this dataset. The solid lines in panels with boxplots connect groups if ANOVA is significant with significance values as: * *p* < 0.05, ** *p* < 0.01, or *** *p* < 0.001.

To evaluate the effect of anemia on the meconium microbial alpha diversity (within sample diversity), the number of microbial species observed in each meconium sample (richness) and the Shannon index (diversity index determining both the richness and evenness) at the species level were computed. The anemic and control samples were classified based on their socio-demographical parameters into urban and rural. Microbiota Chao-1 richness was significantly different between control urban and rural groups. The Shannon index did not exhibit significant change among the four groups (Fig. 1A). Functional diversity was analyzed using KEGG orthologs and MetaCyc pathway abundances showed also no significant difference in the case of richness and Shannon entropy (Fig. 1B&C). This observed low bacterial diversity can be attributed to the nature of the samples i.e. meconium as reported previously^29^. Sample dissimilarity was determined using different beta-diversity measures like Bray-Curtis distance for compositional changes (Fig. 1D), phylogenetic changes whilst taking into account the abundances of ASVs were estimated via Weighted Unifrac distance (Fig. 1E), metabolic changes were calculated through Hierarchical Meta-Storms (Fig. 1F). Permutational multivariate analysis of variance (PERMANOVA) revealed no significant difference between the anemic and control groups in composition and metabolic function (Fig. 1D&F). PERMANOVA analyses showed 14% variability in the phylogeny between the control urban and rural samples. Figure 1G showed the 10 most abundant genera. A significant increase in *Pseudomonas* abundance was observed in rural control samples as compared to rural anemic samples. *Enterococcus* was found predominantly in control urban populations as compared to anemic urban settings. The low abundance of *Enterococcus* in anemic samples may make them susceptible to pathogenic microbes as they have an impact on the human immune system^30^.

## Key genera associated with sources of variability

**Fig. 2:**
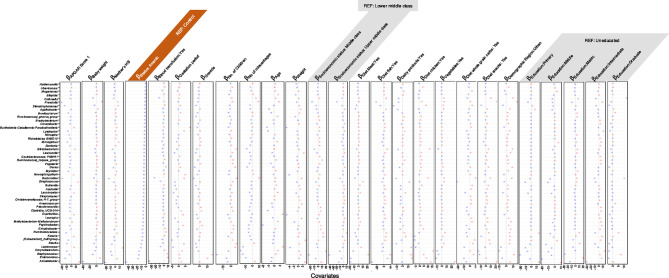
$\:\varvec{\beta\:}-$coefficients returned from GLLVM procedure for model 1 with metadata covariates. Those coefficients which are positively associated with the microbial abundance of a particular species are represented in red color whilst those that are negatively associated are represented with blue color, respectively. Where the coefficients are non-significant, i.e., the 95% confidence interval crosses the 0 boundary, they are greyed out. Since the collation of ASVs was performed at Genus level, all those ASVs that cannot be categorized based on taxonomy are collated under “Unknown” category. For categorical variables, one level acts as a reference and is represented by REF. Taxa for which no taxonomic information was available at genus level were clumped together as *Unknowns* Note that the results are split across three figures with the remaining genera shown in Figs. 3 and 4, respectively.

**Fig. 3:**
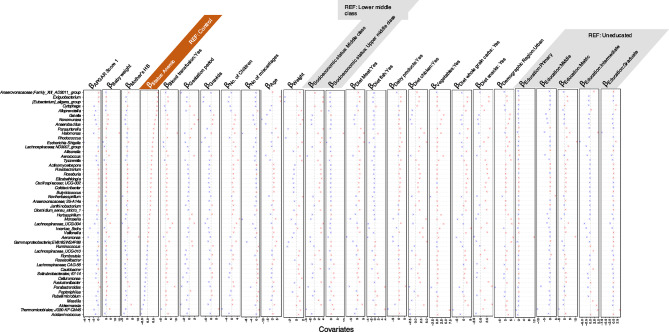
Continuation of results for $\:\varvec{\beta\:}-$coefficients for various covariates returned from the GLLVM procedure for model 1 for metadata, and for the remaining genera not shown in Figs. 2 and 4. Taxa for which no taxonomic information was available at genus level were clumped together as *Unknowns.*

**Fig. 4:**
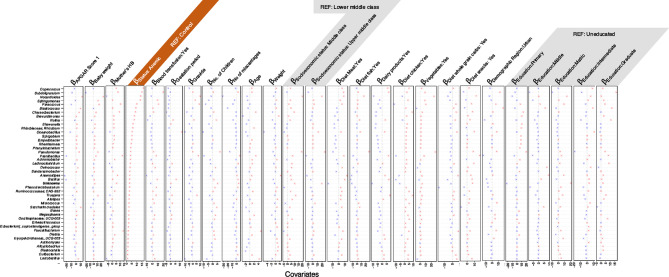
Continuation of results for $\:\varvec{\beta\:}-$coefficients for various covariates returned from the GLLVM procedure for model 1 for metadata, and for the remaining genera not shown in Figs. 2 and 3. Taxa for which no taxonomic information was available at genus level were clumped together as *Unknowns.*

The ASVs were then collated at the genus level and used in the Generalized Linear Latent Variable (GLLVM) to regress their abundances against the observed sources of variability. The returned $\:\beta\:-$coefficients whether positive or negative were then used to assess the associations. The covariates considered in GLLVM include APGAR [Appearance, Pulse, Grimace (reflex irritability), Activity (muscle tone), Respiration] score, baby weight, age of the mother, weight of the mother, hemoglobin level of the mother, blood transfusion, anemic status (normal, anemic), gestation period, gravida, number of children, number of miscarriages, socioeconomic status of the mother (middle and upper middle class), dietary parameters (consumption of chicken, dairy products, fish, snacks, vegetables, whole grain carbs), demographic region (urban, rural), educational status (primary, middle, matric, intermediate, graduate) (Figs. 2, 3 and 4).

Our results indicated that dietary consumption modulates microbial abundance and impacts iron homeostasis. Consumption of a balanced diet comprising of red meat, fish, and dairy- enriched facultative anaerobic bacteria like *Staphylococcus* and *Enterococccus*, a diet devoid of fish consumption and strong association with dairy consumption increased the abundance of anaerobic bacterial genera such as *Coprococcus*,* Subdoligranulum*,* Nocardioides*,* Anaerovoracaceae*,* Exiguobacterium*, and *Eubacterium* that are positively associated with anemia, Exclusive red meat consumption enriched the fermenter bacteria like *Halomonas and Anaerostipes* exhibiting positive correlation with the baby weight, blood transfusion, and maternal weight respectively. Whole grain carbs consumption increased the abundance of commensal bacteria like *Lactobacillus*, displaying a negative association with maternal hemoglobin levels whereas *Bacteroides*, *Faecalibacterium*,* Incertae-Sedis* exhibited a positive association with the number of children, miscarriages maternal weight, and mother hemoglobin level. Chicken and vegetable consumption increased the abundance of pathogenic *Acinetobacter* that correlated positively with miscarriages and maternal weight.

### Association of microbial diversity with SCFA

**Fig. 5:**
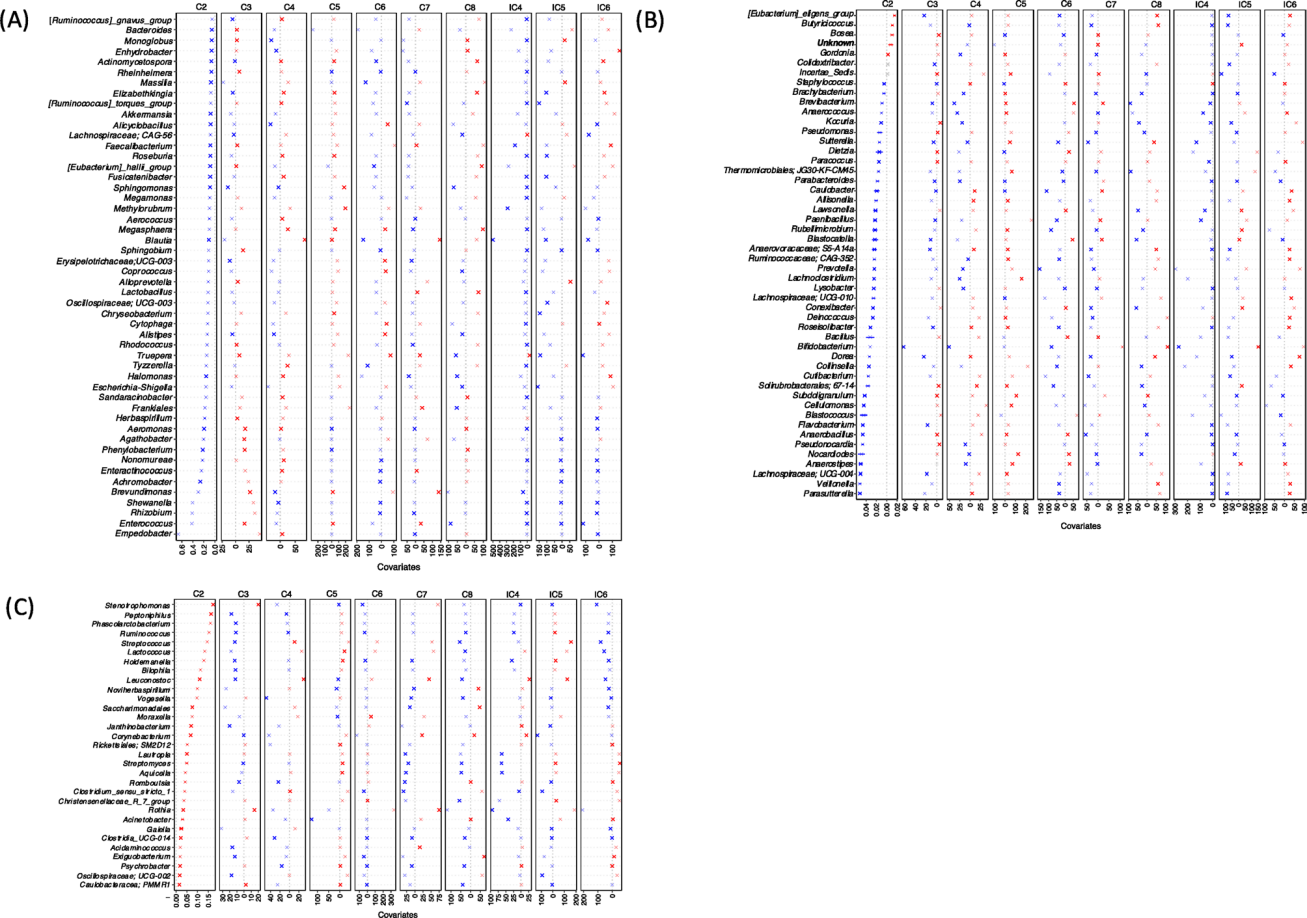
$\:\varvec{\beta\:}-$coefficients returned from GLLVM procedure for model 2 with SCFA covariates. Those coefficients which are positively associated with the microbial abundance of a particular species are represented in red color whilst those that are negatively associated are represented with blue color, respectively. Where the coefficients are non-significant, i.e., the 95% confidence interval crosses the 0 boundary, they are greyed out. Since the collation of ASVs was performed at Genus level, all those ASVs that cannot be categorized based on taxonomy are collated under “Unknown” category. The results are split across three panels. .

GLLVM was applied the second time to identify the association of microbial abundances with different short-chain fatty acids. Redundancy analysis using PERMANOVA showed significant changes in acetate (C2) SCFA estimated by Bray-Curtis, Weighted Uni-Frac, and Hierarchial Meta-Storms whereas octanoic acid (C8) differed significantly between the control and anemic groups based on Unweighted Uni-Frac (Table 2). GLLVM analysis revealed enrichment of branched short-chain fatty acids (BSCFA) in the anemic meconium samples. The majority of the bacterial genera such as *Coprococcus*,* Anaerovoracaceae*, and *Exiguobacterium* associated with anemia, are positively associated with isopropyl-hexanoate (IC6) whereas *Eubacterium halii* group associated with iso-valerate (IC5). Bacterial genera such as *Subdoligranulum* and *Nocardioides*, associated with anemia displayed negative correlation with acetate (C2) production. Moreover, the microbial genera including *Acinetobacter*,* Bacteroides*, *Prevotella* and *Faecalibacterium* associated with miscarriages were also branched short-chain fatty acid (BSCFA) producers e.g. iso-valerate (IC5) and iso-caproic (IC6). These genera were enriched in samples where the mothers were consuming mainly whole grain carbs and snacks respectively and displayed a negative association with acetate (C2). The acetate (C2) and butyrate (C4) producing *Lactococcus* (dairy consumption) was positively associated with maternal hemoglobin level whereas octanoate-producing *Lactobacillus* (whole-grain carbs consumption) negatively correlated with the maternal hemoglobin level. Microbial genera that positively associated with gravida such as *Staphylococcus*,* Enterococcus* (red meat, fish, and dairy) and *Thermomicrobiale* were mainly propionate (C3) and iso-valerate (IC5) producers and showed negative correlation with iso-caproic (IC6) and acetate (C2) levels. Exclusive red meat consumption leads to the enrichment of isopropyl-hexanoate (IC6) and hexanoate (C6) producing bacteria such as *Halomonas* and *Anaerostipes*. Microbial genera such as *Bifidobacterium* (chicken and vegetables consumption) *and Streptococcus (*dairy products consumption) are positively associated with the APGAR score. However, *Bifidobacterium* displayed a positive association with heptanoate (C7), octanoate (C8), iso-valerate (IC5), iso-caproate (IC6), and decreased production of acetate (C2). This data indicated that a balanced diet comprising red meat, fish, and dairy leads to the production of SCFAs such as acetate (C2) and propionate (C3) (Fig. 5).

## Discussion

Although Iron is required in smaller amounts, it plays a critical role in diverse biological processes like oxygen transport, energy production, cell proliferation, and DNA synthesis^31^. Iron levels are maintained via a tight regulatory mechanism. Intestinal absorption is the key step in iron homeostasis^32^. Different mechanisms underlay the host-microbiome crosstalk, including changes in microbial diversity and the production of metabolites^33^. The present study aims to identify key clinical and sociodemographic parameters associated with anemia, based on a questionnaire survey. Our results demonstrated dynamic changes in the gut microbiome and SCFAs during IDA. These findings are mainly attributed to dietary factors. The lack of red meat and fish consumption showed a strong correlation with the incidence of anemia. Furthermore, high cow/buffalo milk consumption was also positively associated with IDA. Low red meat consumption has been previously associated with anemia in children^34^. Elderly males in the Japanese population with higher protein intake particularly fish had lower anemia prevalence^35^. Milk consumption paused a 1.6 times higher risk of moderate anemia probably due to the dilutive effect of milk on dietary iron or its interference with iron absorption. Previous studies have highlighted iron malabsorption due to cow milk consumption^36,37^. Upset plot has demonstrated that dietary pattern significantly affect the haemoglobin levels. Individuals on balanced diet had normal Hb levels whereas the individuals solely on chicken and vegetables consumption tend to be anemic. These findings are in line with other studies demonstrating the impact of diet on iron absorption and haemoglobin levels^38,39^. The previous report in India demonstrated that the B-blood group is anemia prone and the O blood group is anemia resistant^40^. In our study AB- blood group individuals had a relatively high risk of anemia as compared to other blood groups. This signifies that ethnic diversity can change the propensity of blood groups towards anemia susceptibility. This finding is in line with a previous study demonstrating that the high calcium, casein content, and low levels of vitamin C in cow milk can reduce iron intestinal absorption^41^. Previous studies have also documented that diet may influence gut microbiome functioning^42^. The sociodemographic factors also played a pivotal role in microbial abundance primarily due to dietary habits and environmental factors. Among the clinical parameters, hematological parameters HCT, MCV, and MCH appeared to predict anemia. HCT was the most promising predictor of anemia in the Pakistani population. Different studies have highlighted low MCV levels in anemic individuals^12,43^. Lack of balanced diet consumption led to the incidence of moderate and severe anemia. Our results also showed iron deficient diet or malabsorption of iron resulting in a paradigm shift in the gut microbial diversity which may lead to the host susceptibility towards pathogenic bacteria and a decrease in the abundance of beneficial bacteria like *Enterococcus*. Moreover, microbiome and SCFAs analysis also revealed the enrichment of branched SCFAs (BSCFAs) producing anaerobic bacteria such as *Coprococcus*,* Anaerovoracaceae*, and *Exiguobacterium* in IDA patients, along with the decreased presence of acetate and propionate. *Coprococcus*,* Anaerovoracaceae*, and *Exiguobacterium* are negatively associated with fish consumption. BSCFAs (isovalerate, iso-butyrate, and 2-methyl butyrate) are produced due to the fermentation of undigested proteins comprising leucine, isoleucine, and valine-like amino acids reaching colon. Previous study has reported that intestinal dysbiosis due to iron deficiency anemia leads to increased butyrate (C4) level^16^. The generation of BSCFA is mainly due to the fermentation of undigested protein in the colon^44^.To our knowledge, this is the first study that revealed BSCFA’s association with anemia. The production of BSCFA is attributed mainly to protein fermentation and decreased availability of fermentable carbohydrates^44^. A recent study has also documented that during IDA the carbohydrate metabolism shifts towards monosaccharides processing through amino sugar and nucleotide pathway. These BSCFAs have a profound impact on lipid and glucose metabolism^44^ and their elevated levels may damage colon epithelium^45^. Fecal-omic data from preterm severe anemic infants showed its association with pro-inflammatory gut harboured with bacteria of increased virulence potential and decreased metabolic activity of commensal lactic acid producing bacteria^46^.

These findings highlight how dietary factors can shape the gut microbial diversity along with its impact on different metabolic disorders. In order to combat the impact of microbial dysbiosisin the gut different dietary modification like fibre intake, drugs like statins or probiotic like *Lactobacillus reuteri* supplementation has been documented recently^47,48^. However, future studies are required to determine the impact of different probiotics on the gut dysbiosis implicated in the case of anemia. Further studies are required to elucidate the relationship between BSCFA and anemia. Moreover, it will be interesting to see how these BSCFA-producing bacteria during IDA can be used to tackle local and systemic disease-derived alterations leading to improvements in disease management.

### Methods

#### Ethics statement

This study was approved by the Ethics Review Board (ERB) at COMSATS University Islamabad (CUI/Bio/ERB/2021/60). All participants provided written informed consent to participate in the study, and to use the data in research publication. The research involving human participants was performed in accordance with the Declaration of Helsinki.

All experiments were performed in accordance with relevant guidelines and regulations approved by the COMSATS Ethics review board.

### Study area and participants selection

A research project was carried out in Rawalpindi, Pakistan, involving a group of 425 pregnant women who received care at the District Headquarter Hospital (DHQ). Information was gathered from the perinatal database, which records data systematically based on set guidelines immediately following childbirth. This process involved coding, which was conducted after reviewing medical records of prenatal care and standard hospital documentation.

All pregnant females were pre-screened and recruited through a questionnaire, based on inclusion/exclusion criteria as follows. Only those pregnant females were recruited who underwent caesarean section. Pregnant females taking antibiotics within the last three months, who have chronic infections or Hepatitis, were excluded from the study.

### Questionnaire design and data collection

Based on expert consultation and literature survey, a broad-range questionnaire was drafted consisting of ~ 30 questions (Supplementary Material 1). The meconium samples were taken from new-borns delivered via caesarean section. The new-borns delivered through vaginal birth were excluded based on the microbiome differences previously reported due to mode of delivery^49^. For each participant, information was collected on various demographic aspects such as ethnicity, level of education, their place of residence (urban or rural) and dietary habits. We have recorded maternal age, the number of pregnancies (gravidity), number of births (parity), instances of abortion, occurrences of twin pregnancies, medical history including conditions like diabetes mellitus and hypertension, if any blood transfusions received before delivery, and iron supplementation. Moreover, maternal and perinatal outcomes, such as early labour (identified as childbirth occurring before 37 full weeks of pregnancy), bleeding after childbirth, death of the mother, Apgar scores (a quick test to evaluate the health of the new-born at 1 and 5 min after birth), the new-borns’ weight, whether the new-born was admitted to the neonatal intensive care unit (NICU), and perinatal death, have also been recorded.

### Sample collection for faecal microbiome analysis

42 new-born babies born through caesarean section including 31 Iron deficiency anemia (IDA) patients and 11 healthy subjects, were recruited. The IDA patients were diagnosed according to World Health Organization (WHO) diagnostic criteria. Informed consent was obtained from the parents of all individual participants. A detailed questionnaire (previously described) has been filled out for each participant, separately. Fresh meconium samples were collected from diapers and immediately frozen at −20^o^C. Samples were delivered to Microbiology and Public Health Laboratory, COMSATS University Islamabad, where they were stored at −80^o^C until processing.

### DNA extraction, total microbial load and sequencing

Total bacterial load (log10 number of 16 S rRNA gene copies per gram of faeces) was assessed via quantitative PCR carried out on a 7500 Real-Time PCR system (Applied Biosystems, Carlsbad, CA) and performed using TaqMan chemistry (Fisher Scientific), bovine serum albumin (20 mg/mL), 2.5 µM Taqman probe (FAM reporter, BHQ-1 Quencher, CTT GTA CAC ACC GCC CGT C), DNA template diluted 1:100, and the universal reverse (TAC GGC TAC CTT GTT ACG ACT T) and forward (CGG TGA ATA CGT TCC CGG) 9 µM primers. Five serial dilutions of Bacteroides vulgatus genomic DNA obtained from a pure culture were used as standards.

For DNA extraction, DNeasy PowerSoil Pro Kits were used according to manufacturer’s specifications. DNeasy PowerSoil Pro kit was used as it is documented in different studies to yield good quality and high DNA yield^50–52^. 16 S rRNA sequencing of the V4 region was performed on an Illumina MiSeq platform (Illumina, UK) using the Schloss 250 bp paired end method and utilising the primers given in^53^.

### Quantification of SCFAs with gas chromatography

Faecal SCFAs were measured using gas chromatography-flame ionisation detector (GC-FID) (Agilent 7890 A GC, Agilent Technologies, USA). Faecal slurries (1:1 w/v) of 1 g of faecal material and 1 ml of NaOH were prepared at the day of initial stool sample processing and were stored at −20^o^C after vigorous vortexing in the presence of glass beads. Faecal slurries were lyophilised for 36 h in an Edwards Micro Modulyo freeze dryer and the lyophilised samples were then homogenised. 100 mg of lyophilised faecal material was accurately weighed and mixed with 300µL of water in 15 mL tubes. 100µL of an in-house prepared internal standard solution (2-ethylbutyric acid, 73.6 mM) was added to the sample, immediately followed by 100µL of concentrated orthophosphoric acid to lower the dissociation constant and release the acids into the ether phase. The mixture was thoroughly extracted 3 times using 1.5 mL of diethyl ether on an orbital shaker for 1 min each time. The supernatant (ether phase) was recovered and pooled. 1µL of the pooled extract was injected splitless to an Agilent 7890 A GC. Nitrogen was used as the carrier gas. An external standard mixture of SCFAs of known concentrations was used for the calibration curves. This standard consisted of acetate (172.9 µM), propionate (133.7 µΜ), isobutyrate (104.5 µΜ), butyrate (110.2 µΜ), isovalerate (87.5 µΜ), valerate (90.6 µΜ), isocaproic acid (53.3 µΜ), caproic acid (80.2 µΜ). The chromatograph and peak integrals were analysed using the Agilent G2070BA GC ChemStation software (Agilent Analytics, USA). Samples were extracted and analysed in duplicate and in reverse order to correct for any potential time effect during extraction. Results were averaged and repeated if coefficient of variation > 10%.

### Bioinformatics

We have obtained 2,922,419 paired-end reads from 68 samples. On these, we recovered Amplicon Sequencing Variants (ASVs). Briefly, the Dada2 algorithm^54^ within the QIIME2^55^ platform (version 2019.7.0) was used with the parameters --p-trim-left-f 0 --p-trim-left-r 0 --p-trunc-len-f 225 --p-trunc-len-r 215 in qiime dada2 denoise-paired plugin. This yielded a *n* = 68 (samples) X 1,723 (ASVs) abundance table with summary statistics of sample-wise reads matching to ASVs as follows: [Min:149; 1 st Quartile:14,353; Median:37,957; Mean:35,513; 3rd Quartile: 54,116; Max:82,227]. Additionally within QIIME2 platform: (a) the rooted phylogenetic tree for the ASVs was obtained using the Qiime phylogeny align-to-tree-mafft-fastree plugin; (b) the taxonomic information was obtained for the recent SILVA SSU Ref NR database release v.138^56^ using the qiime feature-classifier classify-sklearn plugin; and (c) the metabolic profiles were obtained using qiime picrust2 full-pipeline (https://github.com/picrust/picrust2/wiki/q2-picrust2-Tutorial/)with the parameters --p-hsp-method pic --p-max-nsti 2.Of 1,723 ASVs, 24 ASVs were excluded as they poorly aligned to the reference genomes, and further 13 ASVs were excluded as they were above the maximum NSTI cut-off of 2.0, thus with 1,686/1,723 (97.9%) ASVs matching to the reference database in PICRUSt2 software^57^ increases our confidence in the returned metabolic predictions. Through PICRUSt2, we have obtained 10,543 KEGG Orthologs (KOs) and 489 MetaCyc pathways for 68 samples.

### Statistics

For the categorical data, to see if any two covariates have a relationship, we constructed a contingency table and used $\:{\chi\:}^{2}$ test of independence using chisq.test() function in R. Based on http://www.sthda.com/english/wiki/chi-square-test-of-independence-in-r, and where the relationship existed, we then calculated $\:{\chi\:}^{2}$ residuals for individual rows and columns of the contingency table. These were drawn using R’s corrplot^58^ package where positive values in cells specify an attraction (positive association; blue) between the corresponding row and column variables whilst negative values implies a repulsion (negative association; red) between the corresponding row and column variables. To get the relative risks for disease outbreak, we have used generalized linear models (GLMs) with log link functions to binomial data using R’s logbin package^59^. To generate the regression tables, we have used tab_model() function from R’s sjPlot package^60^ which also facilitated confidence interval display. In some cases, where we had more than two categories in the outcome variable, we have used multinomial logistic regression using multinom() R’s nnet package^61^ with recommendations given in https://stats.oarc.ucla.edu/r/dae/multinomial-logistic-regression/.

Following the recommendations given at https://docs.qiime2.org/2022.8/tutorials/filtering/, as a pre-processing step, we have removed typical contaminants such as *Mitochondria* and *Chloroplasts*, as well as any ASVs that were unassigned at all levels. Low read samples (< 5000 reads) were filtered out, as well as any samples not part of this study. This gave us a final abundance table of *n* = 28 x *P* = 443 ASVs with the summary statistics of reads mapping to these ASVs for samples as follows: [Min:8,777; 1 st Quartile:24,073; Median:31,363; Mean:31,691; 3rd Quartile:38,442; Max:54,178].

The R’s vegan package^62^ was used for alpha and beta diversity analyses. For alpha diversity measures we have used (after rarefying to minimum library size *Shannon entropy* (a commonly used index to measure balance within a community) and *Chao1 richness* (estimated number of species/features in an abundance table). We have used R’s aov() function to calculate the pair-wise analysis of variance (ANOVA) p-values which were then drawn on top of alpha diversity figures. To visualise the beta diversities, we have used Principal Coordinate Analysis (PCoA) (using cmdscale() function from R’s Vegan package) with different distance measures. Specifically, we have used three different measures in PCoA: (i) *Bray-Curtis distance* on the ASV abundance table to visualise the compositional changes; (ii) *Unweighted/Weighted UniFrac distance* estimated using R’s Phyloseq package^63^ on the rooted phylogenetic tree of the ASVs along with the ASV abundance table to see changes between samples in terms of phylogeny; and (iii) *Hierarchical Meta-Storms* (HMS)^64^ on the KOs abundance table. HMS calculates the functional beta diversity distance in a hierarchical fashion utilising the multi-level KEGG BRITE hierarchy to give a weighted dissimilarity measure. In PCoA plots, ellipses of 95% confidence interval of standard error of groups were plotted using ordiellipse() function from R’s Vegan package.

To select the parameters most strongly associated with the variance of the observed communities, we have also applied redundancy analysis (RDA) on different beta diversity distances using Vegan’s capscale() and ordistep() functions in the following set of commands: cap.env = capscale(abund_table.dist~., meta_table); mod0.env = capscale(abund_table.dist ~ 1, meta_table); step.env = ordistep(mod0.env, scope = formula(cap.env), direction=”both”, Pin = 0.1, perm.max = 9999, R2 scope = TRUE). step.env$anova with *p* < 0.05 was then able to identify the subset of parameter which were later used in the PERMANOVA analysis using adonis2() function from R’s Vegan package. This approach has previously been used in^65^ as a variable selection approach before applying PERMANOVA analysis. For abund_table.dist, we have used different beta diversity distances including Bray-Curtis, Unweighted/Weighted UniFrac, and Hierarchical Meta-Storms. We have used the model twice, once for SCFA analysis, and once for the metadata analysis.

To find the relationship between individual microbial genera (ASVs collated at genus level using SILVA taxonomy), and select metadata variables (particularly those concerned with demographics, and relate to antibiotic usage, knowledge, and consumption, Generalised Linear Latent Variable Model (GLLVM)^66^ was used. GLVMM extends the basic generalized linear model that regresses the mean abundances $\:{\mu\:}_{ij}$ (for $\:i$-th sample and $\:j$-th microbe) of individual microbes against the covariates $\:{x}_{i}$ by incorporating latent variables $\:{u}_{i}$ as $\:g\left({\mu\:}_{ij}\right)={\eta\:}_{ij}={\alpha\:}_{i}+{\beta\:}_{0j}+{\varvec{x}}_{i}^{T}{\varvec{\beta\:}}_{j}+{\varvec{u}}_{i}^{T}{\varvec{\theta\:}}_{j}$, where $\:{\varvec{\beta\:}}_{j}$ are the microbe specific coefficients associated with individual covariate (a 95% confidence interval of these whether positive or negative, and not crossing 0 boundary gives directionality with the interpretation that an increase or decrease in that particular covariate causes an increase or decrease in the abundance of the microbe), and $\:{\varvec{\theta\:}}_{j}$ are the corresponding coefficients associated with latent variable. $\:{\beta\:}_{0j}$ are microbe-specific intercepts, whilst $\:{\alpha\:}_{i}$ are optional sample effects which can either be chosen as fixed effects or random effects. We have run the algorithm twice, once for metadata and once for SCFAs.

For metadata, we have used the following covariates in the procedure (type and values they take are written right next):

- Status (Categorical: Anaemic; Control [REF])
- Mothers’ HB (Continuous).
- Blood transfusion (Categorical: Yes; No [REF])
- Age (Continuous).
- Education (Categorical: Primary; Middle; Matric; Intermediate; Graduate; Uneducated [REF])
- No. of Children (Continuous).
- Demographic region (Categorical: Urban; Rural [REF])
- Weight (Continuous).
- No. of Miscarriages (Continuous).
- Gravida (Continuous).
- Gestation period (Continuous).
- Baby Weight (Continuous).
- APGAR SCORE 1 (Continuous).
- Socioeconomic status (Categorical: Upper middle class; Middle class; Lower middle class [REF])
- Diet chicken (Categorical: Yes; No [REF])
- Vegetables (Categorical: Yes; No [REF])
- Diet fish (Categorical: Yes; No [REF])
- Diet whole grain carbs (Categorical: Yes; No [REF])
- Diet Snacks (Categorical; Yes; No [REF])
- Dairy products (Categorical; Yes; No [REF])

For SCFAs have used the following covariates in the procedure (type and values they take are written right next):

- C2 (Acetic acid).
- C3 (Propionic acid).
- C4 (Butyric acid).
- C5 (Valeric acid).
- C6 (Caproic acid).
- C7 (Heptanoic acid).
- C8 (Octanoic acid).
- IC4 (Isobutyrate).
- IC5 (Isovalerate).
- IC6 (Continuous).

REF refers to a reference that gets dropped in the regression model when coding for categorical parameters. To model the distribution of individual microbes, we have used Negative Binomial distribution. Additionally, the approximation to the log-likelihood is done through Variational Approximation (VA) with final sets of parameters in glvmm() function being family = ‘negative.binomial’, method="VA”, and control.start = list (n.init = 7, jitter.var = 0.1) which converged for both models.

## Electronic supplementary material

Below is the link to the electronic supplementary material.

Supplementary Material 1

Supplementary Material 2

Supplementary Material 3

## Data Availability

The raw sequence files supporting the results of this article are available in the European Nucleotide Archive under the project accession number PRJEB77861 available at https://www.ncbi.nlm.nih.gov/bioproject/?term=PRJEB77861 with details given in Supplementary Material 2. Additional annotation for the samples is provided through a survey with the questionnaire used as Supplementary Material 1.
