# Supplementary material for: Gut microbial ecology and function of a Pakistani cohort with Iron deficiency Anemia
Source: Sci Rep. 2025 May 20;15:17532. doi: 10.1038/s41598-025-02556-0 (PMC12092841; doi:10.1038/s41598-025-02556-0)
Supplement: Supplementary file 1 — Supplementary Material 1 [file 41598_2025_2556_MOESM1_ESM.docx]

Supplementary Questionnaire

1. **BIOGRAPHICAL DATA**

Name:

Phone no:

Age:

Gender:

Marital Status:

Patient ID:

1. **EXCLUSION CHECKLIST (If you answer ‘Yes’ to any question, please do not proceed with the questionnaire)**

| # | **Question** | Yes | No |
| --- | --- | --- | --- |
| 1 | Have you taken antibiotic in last 2 months? |  |  |
| 2 | Have you suffered from any disease like Hepatitis, Cancer, TB etc? |  |  |
| 3 | Have you undergone normal delivery? |  |  |

1. **MOTHER’S HISTORY**

| # | **Question** |  |
| --- | --- | --- |
| 1 | Weight of mother |  |
| 2 | Mother’s HB |  |
| 3 | Serum ferritin |  |
| 4 | Gravida |  |
| 5 | Blood group |  |
| 6 | No of children |  |
| 7 | No of miscarriages |  |
| 8 | Gestation period |  |
| 9 | Years of marriage |  |
| 10 | Level of HCT in blood |  |
| 11 | Level of MCV in blood |  |
| 12 | Level of MCH in blood |  |
| 13 | Level of MPV in blood |  |
| 14 | Level of Platelets in blood |  |

1. **INFANT’S HISTORY**

| # | **Question** |  |
| --- | --- | --- |
| 1 | Gender of baby |  |
| 2 | Cord blood hb |  |
| 3 | Baby’s weight |  |
| 4 | APGAR SCORE 1 |  |
| 5 | APGAR SCORE 2 |  |

1. **DEMOGRAPHIC INFORMATION**

**1.** **Patient’s Status**:

a) Normal b) Anemic

**2**. **Gender**

a) Male b) Female c) Other (Please specify):­­­­­­­­­­­­­­­­­­__________________

**3**. **Blood transfusion before delivery?**

a) Yes b) No

**3.      Highest level of education:**

a) Uneducated b) Primary c) Matric d) Intermediate e) Graduate

**4**. **Occupation**

a) Housewife b) Working

**5**. **Demographic region**

a) Urban. b) Rural

**6**. **Use of contraception?**

a) Yes b) No

**7**. **Menstrual cycle**

a) Regular b) Irregular

**8**. **Socio-economic status**

a) Middle class b) Lower middle class. c) Upper middle class

1. **HEALTH CONDITIONS**

Do you have or have ever been diagnosed with following conditions? (Please encircle Yes or No for each)

1. Hypertension: Yes/No
2. Diabetes: Yes/No
3. Uric acid: Yes/No
4. Thrombocytopenia: Yes/No
5. Beta-thalassemia: Yes/No
6. Jaundice: Yes/No
7. Hepatitis A: Yes/No
8. Hepatitis B: Yes/No
9. **DIETRY HABITS**

| # | **Question** | Yes | No | N/A |
| --- | --- | --- | --- | --- |
| 1 | Do you eat chicken regularly? |  |  |  |
| 2 | Do you eat beef regularly? |  |  |  |
| 3 | Do you eat vegetables regularly? |  |  |  |
| 4 | Do you eat fish at least 3 days each week? |  |  |  |
| 5 | Do you consume whole grain carbs regularly? |  |  |  |
| 6 | Do you consume dairy products every day? |  |  |  |
| 7 | Do you consume snacks at least 3 days each week? |  |  |  |
