# Supplementary material for: Gut microbial ecology and function of a Pakistani cohort with Iron deficiency Anemia
Source: Sci Rep. 2025 May 20;15:17532. doi: 10.1038/s41598-025-02556-0 (PMC12092841; doi:10.1038/s41598-025-02556-0)
Supplement: Supplementary file 3 — Supplementary Material 3 [file 41598_2025_2556_MOESM3_ESM.docx]

**Supplementary Materials**

**Supplementary Table S1**: Major risk predictors associated with Anemia status by fitting multinomial regression model. The significant predictors that cause an increase in risk, are shown as **bold**, whilst those that cause a decrease in risk are shown as underlined.

| Outcome | | Parameters | Factors | Risk ratio | p-value |
| --- | --- | --- | --- | --- | --- |
| Normal (REF) | |  |  |  |  |
| Moderate anaemic | |  |  |  |  |
|  | | Intercept |  | 0.2657026 | 0.5500921 |
|  | | Age |  | 0.9739067 | 0.3732344 |
|  | | Marriage Duration |  | 1.021920 | 0.7026133 |
|  | | Gravida |  | 1.1190637 | 0.4321203 |
|  | | Age of menarche |  | 1.006712 | 0.9046383 |
|  | | Miscarriage |  | 0.06554196 | 0.2255640 |
|  | | Blood transfusion before delivery | No (REF) |  |  |
|  | |  | **Yes** | **9.800701***** | **5.736058e-06** |
|  | | Education | Uneducated (REF) |  |  |
|  |  | | **Primary** | **2.500439e+00*** | **0.01085237** |
|  | |  | **Secondary** | **2.292280*** | **0.01200739** |
|  | |  | Higher secondary | 2.1763826 | 0.1062612 |
|  | |  | **Bachelors** | **3.242215e+00*** | **0.03869564** |
|  | | Socio economic status | Lower Middle Class (REF) |  |  |
|  | |  | Upper Class | 1.126947e+00 | 0.9113577 |
|  | |  | Middle class | 0.9141684 | 0.7825196 |
|  | | Diet Fruits | No (REF) |  |  |
|  | |  | Yes | 1.3167338 | 0.3671634 |
|  | | Diet Vegetables | No (REF) |  |  |
|  | |  | Yes | 0.5170759 | 0.2078062 |
|  | | Diet Chicken | No (REF) |  |  |
|  | |  | Yes | 2.066831 | 0.1316669 |
|  | | Diet Fish | No (REF) |  |  |
|  | |  | Yes | 2.366273e-01 | 0.004992683 |
|  | | Diet Red meat | No (REF) |  |  |
|  | |  | Yes | 0.3556019** | 0.001435779 |
|  | | Diet Milk | No (REF) |  |  |
|  | |  | **Yes** | **1.6375720**** | **0.1040162** |
|  | | Blood Group | O+ (REF) |  |  |
|  | |  | A+ | 1.262272 | 0.501298 |
|  | |  | A- | 3.703555e-01 | 0.1943467 |
|  | |  | AB+ | 0.4622176 | 0.1542051 |
|  | |  | **AB-** | **1.262660e+07***** | **<0.001 ***** |
|  | |  | B- | 0.5337550 | 0.2779849 |
|  | |  | B+ | 1.070353 | 0.8345489 |
|  | |  | O- | 0.4643503 | 0.3784923 |
|  | | Disease Hypertension | No (REF) |  |  |
|  | |  | Yes | 1.576971e-01 | 0.09600505 |
|  | | Disease Diabetes | No (REF) |  |  |
|  | |  | Yes | 5.930259e+00 | 0.1007662 |
|  | | Gestation | Single (REF) |  |  |
|  | |  | Twin | 2.229153e-01 | 0.09465044 |
|  | | Gender of baby | Female (REF) |  |  |
|  | |  | Male | 1.231061 | 0.4126391 |
|  | | Baby weight |  | 0.9599187 | 0.88950471 |
|  | | APGAR score 1 |  | 0.9144582 | 0.7952618 |
|  | | APGAR score 2 |  | 1.2563843 | 0.5267419 |
| Severe Anaemic | | Intercept |  | 9.0937482 | 0.7727582 |
|  | | Age |  | 0.8509530 | 0.2114815 |
|  | | Marriage Duration |  | 1.180706 | 0.4273845 |
|  | | Gravida |  | 0.7053772 | 0.4935800 |
|  | | Age of menarche |  | 1.086104 | 0.6987957 |
|  | | Miscarriage |  | 2.2252190 | 0.2506758 |
|  | | Blood transfusion before delivery | No (REF) |  |  |
|  | |  | **Yes** | **137.325211***** | **1.346987e-05** |
|  | | Education | Uneducated (REF) |  |  |
|  | |  | Primary | 2.338287e-13*** | <0.001 *** |
|  | |  | Secondary | 1.444197 | 0.70999287 |
|  | |  | Higher secondary | 0.7805057 | 0.9072013 |
|  | |  | Bachelors | 3.185924e-07*** | <0.001 *** |
|  | | Socio economic status | Lower Middle Class (REF) |  |  |
|  | |  | Upper class | 3.973140e-08*** | <0.001 *** |
|  | |  | Middle class | 0.2049013 | 0.1611034 |
|  | | Diet Fruits | No (REF) |  |  |
|  | |  | Yes | 0.3374324 | 0.2633337 |
|  | | Diet Vegetables | No (REF) |  |  |
|  | |  | Yes | 0.9781175 | 0.9899292 |
|  | | Diet Chicken | No (REF) |  |  |
|  | |  | Yes | 1.601598 | 0.7869125 |
|  | | Diet Fish | No (REF) |  |  |
|  | |  | Yes | 7.863172e-08*** | <0.001 *** |
|  | | Diet Red meat | No (REF) |  |  |
|  | |  | Yes | 0.3056836 | 0.453932374 |
|  | | Diet Milk | No (REF) |  |  |
|  | |  | Yes | 0.9072113 | 0.9208771 |
|  | | Blood group | O+ (REF) |  |  |
|  | |  | A- | 2.501112e-08*** | <0.001 *** |
|  | |  | A+ | 9.176033 | 0.1180767 |
|  | |  | AB+ | 5.5347051 | 0.2977084 |
|  | |  | **AB-** | **3.019907e+00***** | **<0.001 ***** |
|  | |  | B- | 0.4748651 | 0.6898719 |
|  | |  | B+ | 5.270759 | 0.2240047 |
|  | |  | O- | 0.7286989 | 0.8887269 |
|  | | Disease Hypertension | No (REF) |  |  |
|  | |  | Yes | 1.863299e-05*** | <0.001 *** |
|  | | Disease Diabetes | No (REF) |  |  |
|  | |  | Yes | 2.782256e-05*** | <0.001 *** |
|  | | Gestation | No (REF) |  |  |
|  | |  | Twin | 3.278934e-07*** | <0.001 *** |
|  | | Gender of baby | Female (REF) |  |  |
|  | |  | Male | 2.145835 | 0.3826295 |
|  | | Baby weight |  | 0.2151986 | 0.08554871 |
|  | | APGAR score 1 |  | 2.6577758 | 0.3243259 |
|  | | APGAR score 2 |  | 0.4535603 | 0.4279115 |
| Observations | | 382 |  |  |  |
| R^2^/R^2^ adjusted | | 0.238/0.235 |  |  |  |

**Supplementary Figure S1**: **Significant dependences recovered between categorical-response questions in self-reported questionnaire using 𝜒^2^ test of independence**. Where significant (p<0.05), the 𝜒^2^ Pearson residuals are calculated. The positive value represented by the blue color, is a positive attraction between the corresponding row and column variables whilst negative values imply a repulsion (negative association; red) between the corresponding row and column variables.

**Supplementary Figure S2**: **Comparison of key parameters for different groups with Iron Deficiency Anemia**. After autoscaling the data, the non-parametric Kruscal Wallis test was performed with p-values adjusted for multiple comparisons using Benjamini & Hochberg (1995). The initial parameters list was: Marriage_duration; Gravida; Miscarriage; APGAR_score_1; APGAR_score_2; HCT; MCV; MCH; PLT; and MPV. The final list of parameters (HCT, MCH, and MCV) with adjusted p-values <0.05 are shown in (A). Afterwards, a Random Forest Classifier is fitted on these parameters. Two important measures, (B) mean decrease in Accuracy, and (C) Mean Decrease Gini ranks the features in terms of decreasing importance, i.e., the most important features that segregate between different conditions are shown on the left. To see the performance of the classifier the confusion matrix is shown in (D) (86.36% accuracy) where the rows represent the original labels, and the columns represent the predicted labels by the classifier.

**Supplementary Figure S3: UpSet plot representing the correlation of anemia status with dietary habits.**

**Other supplementary files:**

**Supplementary_Data_Table_S2.xlsx:** Additional metadata file associated with the microbiome samples as well as survey data provided as separate sheets.

**Supplementary_Questionnaire.docx:** Additional questionnaire file used for survey data.
